# Additive effect of a single intravenous dose of acetaminophen administered at the end of laparoscopic hysterectomy on postoperative pain control with nefopam and fentanyl-based patient-controlled analgesia: a double-blind, randomized controlled trial

**DOI:** 10.1186/s12871-025-02971-w

**Published:** 2025-02-20

**Authors:** Seungpyo Nam, Seokha Yoo, Sun-Kyung Park, Jin-Tae Kim

**Affiliations:** 1 Department of Anesthesiology and Pain Medicine, Seoul National University Hospital, Seoul National University College of Medicine, 101 Daehak-Ro, Jongno-Gu, Seoul, 03080 Korea; 2 Department of Anesthesiology and Pain Medicine and Anesthesia and Pain Research Institute, Yonsei University College of Medicine, Seoul, Korea

**Keywords:** Acetaminophen, Fentanyl, Nefopam, Postoperative pain control, Patient-controlled analgesia

## Abstract

**Background:**

Acetaminophen is a widely used analgesic for postoperative pain management. However, data on its combined use with nefopam for managing postoperative pain following laparoscopic hysterectomy are limited. This study evaluated the effects of a single intravenous dose of acetaminophen combined with fentanyl- and nefopam-based patient-controlled analgesia (PCA) in patients undergoing laparoscopic hysterectomy.

**Methods:**

In this prospective, double-blind, randomized controlled trial, 84 patients were randomized to receive either 1 g of intravenous acetaminophen (treatment group, *n* = 42) or normal saline (control group, *n* = 42) at the end of surgery. All patients received fentanyl and nefopam via PCA, postoperatively. PCA consumption, pain scores at rest, and postoperative nausea and vomiting (PONV) scores were assessed at 1, 6, and 24 h postoperatively. Patient satisfaction and opioid-related side effects were also evaluated. The primary outcome was the total PCA consumption within the first 24 h.

**Results:**

No significant difference in 24-h PCA consumption was observed between the control and treatment groups (27.9 ± 16.6 *vs*. 26.4 ± 11.2, *P* = 0.623). The pain scores at rest measured at 1, 6, and 24 h after surgery were also not significantly different between the two groups. There were no differences in the satisfaction scores, PONV scores, rescue analgesic use, adverse effects, or length of hospital stay between the groups.

**Conclusions:**

A single intraoperative dose of intravenous acetaminophen, combined with nefopam- and fentanyl-based PCA, did not significantly reduce analgesic requirements, pain scores at rest, or opioid-related side effects compared with placebo in laparoscopic hysterectomy patients.

**Trial registration:**

ClinicalTrials.gov (Identifier: NCT03644147 | August 21, 2018).

## Background

Hysterectomy is one of the most commonly performed gynecological surgeries [1]. With the adoption of minimally invasive techniques across various surgical practices, laparoscopic approaches have gained preference over traditional abdominal and vaginal hysterectomies in gynecology. Laparoscopic hysterectomy offers several advantages, including reduced postoperative pain and morbidity, faster recovery, and shorter hospital stays [2–4]. However, despite its reputation for being less painful, postoperative pain following laparoscopic hysterectomy can still be quite severe, particularly during the early postoperative period [5, 6]. One study reported that pain levels were most intense during the first postoperative hour, with pain scores exceeding 60 on a 100-mm visual analog scale (VAS) at rest. Although pain typically decreases to less than 40 within 24 h post-surgery, the majority of patients require additional or rescue analgesics during this critical recovery period [5]. While various postoperative pain management protocols are available, there is limited data specifically focused on optimizing recovery following laparoscopic hysterectomy, underscoring the need for further research [7].

Effective postoperative pain management is essential for optimizing recovery and improving patient outcomes. Inadequate pain control can lead to physiological complications and reduce patient satisfaction [8]. Traditionally, systemic opioids have been the primary options for managing severe postoperative pain. However, their use is often limited due to various side effects, including respiratory depression, nausea, vomiting, urinary retention, constipation, and dizziness [9–11]. Consequently, nonopioid medications such as acetaminophen, nonsteroidal anti-inflammatory drugs (NSAIDs), cyclooxygenase-2 (COX-2) inhibitors, and nefopam are increasingly being incorporated into multimodal analgesia strategies to reduce opioid consumption and minimize associated side effects [12, 13]. Among these non-opioid options, the combination of acetaminophen and nefopam has shown promise. A recent network meta-analysis evaluated the efficacy and safety profiles of non-opioid analgesics in major surgeries and found that the combination of acetaminophen and nefopam was superior to most single-agent analgesics in terms of opioid-sparing effects [14]. However, the synergistic effect of acetaminophen and nefopam is supported by limited clinical evidence and remains largely unexplored, particularly in the context of postoperative pain control following laparoscopic hysterectomy.

This study aimed to assess the potential benefits of incorporating a single intravenous dose of acetaminophen into a fentanyl- and nefopam-based patient-controlled analgesia (PCA) regimen for patients undergoing laparoscopic hysterectomy. We hypothesized that the addition of a single intravenous dose of acetaminophen would reduce the total amount of analgesics administered after laparoscopic hysterectomy.

## Methods

### Ethics

This prospective, double-blind, randomized, controlled study was approved by the Institutional Review Board of Seoul National University Hospital (No. 1807–151-961) on August 21, 2018, and was registered on ClinicalTrials.gov before the recruitment of the first participant (NCT03644147, date of registration: August 21, 2018). The study adhered to the CONSORT guidelines, with a completed CONSORT checklist provided as an additional file. The study was conducted in compliance with the Declaration of Helsinki and written informed consent was obtained from all participants before surgery.

### Study population

Subjects were eligible for enrollment if they were between 19 and 80 years old and were scheduled to undergo total laparoscopic hysterectomy under general anesthesia at Seoul National University Hospital. Subjects were excluded if they had an American Society of Anesthesiologists (ASA) physical status greater than II, liver disease (AST/ALT > 80 IU/L), chronic kidney disease (GFR < 30 ml/min/1.73 m^2^), a history of drug allergy, chronic pain lasting more than 3 months, or limitations in expressing pain. Patients were also excluded if the surgery was classified as a complex case requiring collaboration with other experts, or if conversion to laparotomy was necessary.

### Randomization and blinding

Each subject was randomly assigned a sequential study number on the day of surgery to either the intravenous acetaminophen group or the placebo group in a 1:1 ratio. Randomization was performed using R software version 3.4.4 (R Foundation for Statistical Computing, Vienna, Austria), which generates a table of random numbers using block randomization with a randomly selected block size of 2 or 4 in a reproducible sequence. The allocation sequence was securely stored in sequentially numbered, sealed, opaque envelopes to ensure allocation concealment. These envelopes were managed by an independent third party who was not involved in the execution or analysis of the study.

Prior to preparing the patient for surgery, the envelope corresponding to the subject’s study number was opened by a team of three medical personnel unrelated to the study, including a preparation nurse who was responsible for verifying the group assignment and preparing the study medication accordingly. To maintain blinding, the study medication was concealed with opaque material, ensuring that both the patients and attending anesthesiologist remained unaware of the group assignment during administration. Blinding was rigorously maintained throughout the study period and was only lifted after the completion of all required data collection or in case of an emergency or serious drug side effect to ensure patient rights and safety. Outcome assessments were performed by an independent outcome assessor blinded to group allocation to ensure objective and unbiased evaluations.

### Anesthetic and analgesic protocols

General anesthesia induction and maintenance were standardized using total intravenous anesthesia (TIVA) with propofol and remifentanil, targeting a bispectral index (BIS) of 40–60 or a patient state index (PSI) of 25–50, with hemodynamics controlled to a maximum of 20% change from baseline values. During surgical wound closure, the subjects received either 1 g of intravenous acetaminophen (Profa infusion, 1 g/100 mL bottle) or 100 mL of normal saline over 10 min, according to their allocated groups.

Following surgery, all subjects were administered our center’s standardized intravenous PCA regimen. PCA contained a total volume of 100 mL, comprising normal saline, fentanyl 500 µg, and nefopam 80 mg. A loading dose of 4 mL from the PCA device, along with ramosetron 0.3 mg, was administered at the end of the surgery. Upon arrival in the recovery room, the PCA device was connected to the subjects, who were instructed to press the button on the PCA device whenever they experienced moderate or severe pain. The PCA device was programmed to deliver a basal infusion rate of 0.5 mL/h and a bolus dose of 1 mL on demand, with a lockout time interval of 10 min. If subjects required additional rescue analgesics within the lockout interval, intravenous fentanyl 50 µg was administered for a pain score of 7 or above, whereas ketorolac 30 mg was administered for a pain score of 4–6 to manage breakthrough pain. After transfer to the ward, intravenous ketorolac 30 mg or dexketoprofen 50 mg was administered as rescue analgesics at the discretion of the attending physicians upon patient request, and IV PCA was maintained until discharge.

### Outcome variables

The primary outcome of this study was the total volume of drug administered via the PCA device at 1, 6, and 24 h postoperatively. Pain scores at rest and the severity of postoperative nausea and vomiting (PONV) were assessed at the same time points. Pain was measured using an 11-point numeric rating scale (0 = no pain, 10 = worst pain imaginable), while PONV severity was evaluated using a 4-point scale (0 = none, 1 = mild, 2 = moderate, 3 = severe). At 24 h postoperatively, patient satisfaction was assessed using a 0–10 scale (0 = extremely dissatisfied, 10 = extremely satisfied). Additionally, the incidence of respiratory depression, urinary retention, tachycardia, and sweating, the use of rescue analgesics within the first 24 h after surgery, and the total length of hospital stay were recorded.

### Statistical analyses

A preliminary study conducted at our center found that patients undergoing gynecologic surgery received an average of 15 ± 4.6 mL of fentanyl- and nefopam-based PCA during the 24-h postoperative period. Based on previous studies showing that the addition of acetaminophen reduces opioid use by 20–27% within the first 24 postoperative hours [15, 16], we assumed a 20% reduction in PCA consumption with the administration of 1 g of intravenous acetaminophen in our sample size calculation. With an alpha error of 5% and a beta error of 20%, the required sample size was calculated to be 37 patients per group. To account for a 10% dropout rate, a total of 84 patients were included in this study.

Statistical analyses were performed using R software (version 4.2.1) in RStudio (R Core Team, 2022, Vienna, Austria, Version 2023.09.1 + 494). Continuous data are expressed as mean ± standard deviation (SD) or median (interquartile range), as appropriate, while categorical data are summarized as frequencies and percentages.

The total PCA consumption, pain scores at rest, PONV scores, patient satisfaction scores at 24 h postoperatively, and total length of hospital stay were compared between the groups using Student’s t-test or Mann–Whitney U test, as appropriate. Bonferroni correction was applied to adjust for multiple comparisons, including the total PCA consumption, pain scores at rest, and PONV scores measured at each time point. The incidence of adverse effects, including respiratory depression, nausea, vomiting, urinary retention, tachycardia, and sweating, was compared between groups using the chi-square test or Fisher’s exact test, as appropriate. *P* values < 0.05 were considered statistically significant.

## Results

**Fig. 1:**
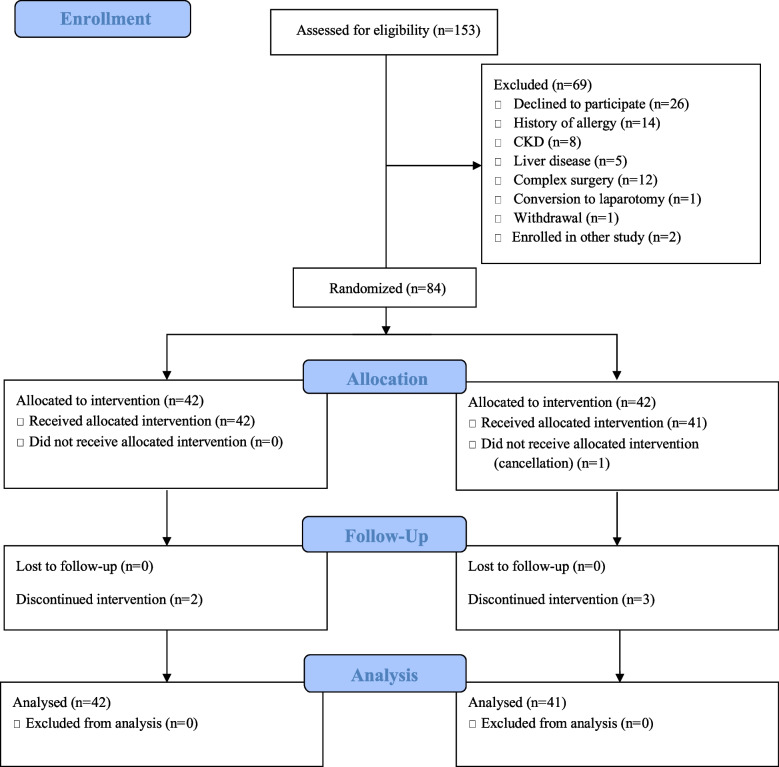
Flow diagram of patient participation

Among the 153 patients screened for eligibility between August 2018 and January 2021, 84 were enrolled; however, one participant from the treatment group withdrew due to surgery cancellation (Fig. 1). No statistically significant differences in demographic characteristics were observed between the two groups (Table 1).

**Table 1:** Demographic data and intraoperative variables

	Control (*n* = 42)	Treatment (*n* = 41)
Age (years)	49 ± 9	48 ± 6
Height (cm)	158.4 ± 5.9	159.4 ± 5.2
Weight (kg)	62.5 ± 11.2	62.4 ± 9.3
BMI (kg/m^2^)	24.9 ± 4.1	24.6 ± 4.0
ASA physical status		
I	31 (73.8%)	29 (70.7%)
II	11 (26.2%)	12 (29.3%)
Anesthetic time (minutes)	80 [70–114]	85 [70–105]
Operation time (minutes)	50 [45–64]	55 [40–75]

*ASA* American Society of Anesthesiologists, *BMI* body mass index

Postoperative PCA consumption and patient assessments over the 24-h postoperative period are summarized in Table 2. Two patients from the control group and three from the treatment group discontinued PCA due to adverse effects during the postoperative period. No significant differences in PCA consumption at 24 h postoperatively were observed between the groups (26.4 ± 11.2 vs. 27.9 ± 16.6, *P* = 0.623; adjusted *P* > 0.999). Similarly, the pain scores at rest were not significantly different between the two groups at each time point (Table 2). Furthermore, no statistically significant differences in satisfaction or PONV scores were found between the groups.

**Table 2:** Postoperative PCA consumptions and patient assessments

	Control (*n* = 42)	Treatment (*n* = 41)	*P*	Adjusted* P*
PCA consumption				
1 h	2.9 ± 1.4	2.3 ± 1.1	0.025	0.075
6 h	12.0 ± 6.1	10.4 ± 4.8	0.611	> 0.999
24 h	27.9 ± 16.6	26.4 ± 11.2	0.623	> 0.999
Pain score at rest				
1 h	6.0 ± 2.0	5.2 ± 1.7	0.041	0.123
6 h	3.2 ± 1.4	3.4 ± 1.6	0.736	> 0.999
24 h	3.1 ± 2.1	2.7 ± 1.5	0.893	> 0.999
PONV score				
1 h	0.2 ± 0.5	0.3 ± 0.6	0.285	0.855
6 h	0.1 ± 0.3	0.4 ± 0.8	0.298	0.894
24 h	0.2 ± 0.4	0.2 ± 0.5	0.286	0.858
Satisfaction score	7.5 ± 2.3	7.9 ± 1.7	0.627	-
Length of stay (days)	4.1 ± 0.4	4.2 ± 0.5	0.697	-

*PCA* patient-controlled analgesia, *PONV* postoperative nausea and vomiting

Over the 24-h postoperative period, approximately 60% of patients in both groups used NSAIDs as rescue analgesics, while approximately 35% of patients in both groups required rescue opioids (Table 3). No statistically significant differences were observed between the two groups in terms of drug-related adverse effects or length of hospital stay.

**Table 3:** Use of rescue analgesics and incidence of adverse effects

	Control (*n* = 42)	Treatment (*n* = 41)	*P*
Rescue analgesics			
NSAIDs	26 (61.9%)	25 (61.0%)	> 0.999
Fentanyl	14 (33.3%)	15 (36.6%)	0.936
Adverse effects			
Respiratory depression	1 (2.4%)	0 (0.0%)	> 0.999
Nausea	15 (35.7%)	15 (36.6%)	> 0.999
Vomiting	1 (2.4%)	4 (9.8%)	0.202
Urinary retention	1 (2.4%)	2 (4.9%)	0.616
Tachycardia	4 (9.5%)	1 (2.4%)	0.360
Sweating	16 (38.1%)	15 (36.6%)	> 0.999

NSAIDs includes ketorolac 50 mg and/or dexketoprofen 50 mg

## Discussion

This prospective, double-blind, randomized controlled study comparing intravenous acetaminophen with placebo at the time of laparoscopic hysterectomy showed no significant difference in PCA consumption or pain score at rest between the groups at any postoperative time point. Additionally, patient satisfaction and the incidence of opioid-related side effects were not different between the groups.

In major surgeries, such as laparoscopic hysterectomy, opioids have traditionally been the cornerstone for effective postoperative pain control. However, their use has become increasingly limited due to adverse effects such as nausea, vomiting, and respiratory depression, shifting the focus toward strategies that reduce pain while minimizing opioid reliance [17, 18]. Consequently, multimodal analgesic approaches combining opioids with non-opioid analgesics have gained popularity for managing postoperative pain [14, 19]. At our center, the addition of nefopam to fentanyl-based IV PCA has been the standard practice for reducing overall opioid usage following laparoscopic hysterectomy [20–28]. A network meta-analysis indicated that combining two non-opioid analgesics, rather than relying on a single agent like nefopam, is more effective in reducing opioid consumption and improving pain relief [14]. Among the various combinations, acetaminophen and nefopam have been identified as one of the most effective pairs for achieving opioid-sparing effects. Acetaminophen, a widely used analgesic with minimal side effects, has proven effectiveness in managing acute and postoperative pain in various procedures, including orthopedic surgery, bariatric surgery, cholecystectomy, cesarean delivery, and abdominal hysterectomy [29–32]. Additionally, a recent preclinical study in rodents demonstrated that acetaminophen may enhance the antinociceptive effects of nefopam [33]. However, despite the hypothesis that adding acetaminophen to a nefopam- and fentanyl-based PCA regimen would enhance efficacy, our study showed no significant improvement in pain scores at rest or opioid-sparing effects compared with placebo following laparoscopic hysterectomy.

There are several possible explanations for these findings. First, a single dose of 1 g intravenous acetaminophen may not provide a sufficient analgesic effect to significantly reduce pain or opioid consumption after laparoscopic hysterectomy. Studies demonstrating the effectiveness of intravenous acetaminophen in reducing pain or opioid usage often involved multiple doses administered at regular intervals rather than a single dose [29–32]. The PROSPECT guidelines recommend prioritizing the use of acetaminophen, NSAIDs, or COX-2 selective inhibitors as primary analgesics [34]. However, supporting evidence for these recommendations is based on a study involving repeated acetaminophen administration, with 1 g given at the induction of anesthesia and every six hours for 24 h, which significantly reduced opioid consumption [35]. In contrast, several previous studies, including our own, have shown that a single dose of intravenous acetaminophen administered postoperatively does not significantly differ from placebo in terms of pain relief or opioid consumption [7, 36]. Therefore, further research is necessary to identify the optimal dosing regimen and timing of acetaminophen administration to maximize its effectiveness in postoperative pain management, particularly in the context of laparoscopic hysterectomy.

An alternative explanation for these findings could be related to the study protocol or design. The PCA regimen used in this study included a basal infusion rate of 0.5 mL/h, which may have contributed to lowering the overall pain scores following laparoscopic hysterectomy. This reduction in baseline pain levels may have potentially masked any additional benefits of the use of intravenous acetaminophen. Furthermore, the sample size calculated for this study may have been underpowered to detect small differences in the PCA consumption. The calculation was based on the assumption that acetaminophen would reduce the PCA consumption by 20%. This assumption was supported by a meta-analysis of randomized controlled trials, which found that adding acetaminophen to PCA morphine resulted in a 20% reduction in morphine consumption during the first 24 postoperative hours following major surgery [15]. Similarly, a randomized controlled trial on cardiac surgery demonstrated a 27% reduction in opioid consumption with intravenous acetaminophen within 24 h postoperatively [16]. However, considering that acetaminophen is a mild non-opioid analgesic with 20–30% less analgesic efficacy than NSAIDs [37], the assumption of a 20% reduction from a single dose of acetaminophen may have been an overestimation.

This study had several limitations. First, as this study was conducted in a single medical center, the results may not be representative or applicable to diverse clinical settings or a broader patient population. In addition, the absence of basal analgesics, such as oral acetaminophen or NSAIDs, during the perioperative period may limit the generalizability of our findings. Second, the outcomes were evaluated only up to 24 h postoperatively. Although this time frame was selected based on the duration of acetaminophen action, a longer observation period extending until discharge could have provided additional insights. Third, this study exclusively evaluated the effects of a single dose of intravenous acetaminophen compared to a placebo following surgery. As the effects of alternative forms, varying dosages, or multiple administrations of acetaminophen were not assessed, it remains unclear whether acetaminophen is truly ineffective for postoperative pain management after laparoscopic hysterectomy. Lastly, the perioperative analgesic regimen in this study does not fully align with the current standard practice, which emphasizes the regular use of non-opioid analgesics, such as scheduled acetaminophen and NSAIDs, with opioids reserved for rescue analgesia rather than PCA opioids [34]. These differences may affect the generalizability of our findings to clinical settings where modern multimodal analgesia is routinely implemented.

## Conclusions

In conclusion, our study found that a single intraoperative dose of intravenous acetaminophen did not significantly reduce pain scores at rest or opioid consumption compared with placebo in patients undergoing laparoscopic hysterectomy. Additionally, no differences were observed in patient satisfaction or the incidence of opioid-related side effects between the groups. Further research is needed to determine the optimal dosage and route of acetaminophen in patients undergoing laparoscopic hysterectomy.

## Data Availability

The datasets used and/or analyzed during the current study are available from the corresponding author on reasonable request.
